# Structure formation in fruit preparations by fruit fermentates produced with exopolysaccharide-forming lactic acid bacteria

**DOI:** 10.1016/j.crfs.2025.101244

**Published:** 2025-11-07

**Authors:** Silvan Festini, Dor Zipori, Marc Wallisch, Agnes Weiss, Sybille Neidhart, Herbert Schmidt, Mario Jekle

**Affiliations:** a University of Hohenheim, Institute of Food Science and Biotechnology, Department of Plant-based Foods, Stuttgart, 70599, Germany; b University of Hohenheim, Institute of Food Science and Biotechnology, Department Food Microbiology and Hygiene, Stuttgart, 70599, Germany; c University of Hamburg, Hamburg School of Food Science, Food Microbiology, Hamburg, 22609, Germany

**Keywords:** Lactic acid bacteria, Rheology, *Levilactobacillus brevis* TMW 1.2112, *Pediococcus parvulus* LTH 1110, Viscosity, Stabilizer, Anion exchange chromatography, Exopolysaccharide, β-glucan

## Abstract

Fruit preparations are intermediate food products that are primarily used in the dairy industry for the production of fruit yogurt or frozen desserts. Typically, they are stabilized by added hydrocolloids like pectins. The objective of this study was to investigate the potential replacement of conventional stabilizers by structure-forming fermentates produced by exopolysaccharides (EPS)-forming lactic acid bacteria (LAB). Peach puree was selected as fermentation matrix. Prior to 72 h of incubation, it was inoculated with either the heterofermentative LAB strain *Levilactobacillus brevis* TMW 1.2112 or the homofermentative LAB strain *Pediococcus parvulu*s strain LTH 1110, both being known to produce EPS in form of β-D-glucan. The lyophilized fermentates were applied as stabilizers to produce strawberry fruit preparations. Flow curves, viscoelastic behaviour and shear stability were measured to investigate the effect of fermentate incorporation on the rheological properties of the products. A fermentatively induced effect was observed in terms of a 1.3-fold increase in viscosity of strawberry model fruit preparations with 10 % fermentate of *Lv. brevis* TMW 1.2112 compared to the addition of the same dose of fermentate blank. Further, increasing the fermentate blank dose from 10 % to 15 % resulted in a 2.4-fold viscosity increase of the model fruit preparations. High shear stability was found in all model strawberry fruit preparations. However, fermentation had no clear benefit in terms of viscoelastic behaviour and shear stability of the fruit preparations. Although the fermentatively induced thickening potential was limited, production of viscosity-increasing peach fermentate with minor changes in the sugar and amino acid profiles of the fruit proved to be feasible.

## Highlights

• LAB-fermented peach puree explored as hydrocolloid alternative.
• Minor changes of chemical composition due to lactic fermentation.
• EPS-producing LAB strains applied in strawberry model fruit preparations.
• Thickening of fruit preparations by incorporating lyophilized peach fermentates.
• Fermentate produced with *Lv. brevis* TMW 1.2112 increased viscosity 1.3-fold.

## Graphical abstract

**Figure:**
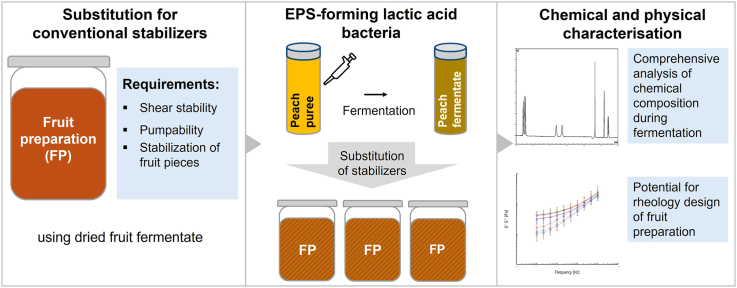

## 1 Introduction

Fruit preparations are intermediate products in the food industry, where they are used in a variety of foods such as the fruit components of dairy products, frozen desserts and bakery goods (Fügel et al., 2006). In commercially available dairy products like fruit yoghurts, fruit preparations typically make up 10–20 % of the volume in the final product. These intermediate products typically consist of at least 35 % fruit puree, added sugars, hydrocolloids as well as coloring agents and aromas (O'Rell and Chandan, 2006). To ensure proper applicability, they must exhibit shear-thinning behavior and shear stability, which allow for pumpability and stirring. Additionally, viscoelastic and thixotropic properties are required to prevent sedimentation or flotation of fruit pieces during processing and storage (Meghwal et al., 2017). To fulfill the requirements set by the food industry, conventionally produced fruit preparations are stabilized by added thickeners in form of hydrocolloids such as pectin or modified starches (O'Rell and Chandan, 2006). However, consumer acceptance of food additives may widely be sparse, especially in Europe, as their E-number declaration is mandatory (Bearth et al., 2014). There is a strong trend towards *clean label* products and the use of the least possible amount of ingredients, and thus towards very limited use of food additives (Asioli et al., 2017). For fruit preparations, it is usual practice to apply coloring foods to improve product color, natural fruit sweeteners and acidic juice concentrates to adjust dry matter, sweetness and acidity, and natural flavoring agents and aroma extracts. However, since thickeners and stabilizers are food additives with highly specific structure-forming efficacy at low doses, their partial or entire substitution by ingredients for *clean label* products is challenging. Hence, there is great demand from the food industry for alternative structure formation solutions.

One promising approach to replace conventional stabilizers and hydrocolloids in food systems involves microbial fermentation with *in situ* production of exopolysaccharides (EPS). EPS are long-chain polysaccharides synthesized and secreted by microorganisms (Zannini et al., 2016). Among them, lactic acid bacteria (LAB) have a long history of safe use as starter cultures in food fermentation and are generally recognized as safe (Zannini et al., 2016). Certain LAB strains can produce various types of EPS, which are broadly classified into homopolysaccharides (HoPS), composed of a single type of carbohydrate monomer, and heteropolysaccharides (HePS), consisting of multiple carbohydrate monomers (Kaur et al., 2020; Ruas-Madiedo et al., 2002). Particularly promising HoPS for the application in fruit preparations are β-glucans due to their heat stability in acidic environments. Their main chains consist of D-glucopyranosyl monomers linked by β-1,3 glycosidic bonds and carry occasional side chains of β-1,2-attached monomeric glucose (Dueñas-Chasco et al., 1997; Garai-Ibabe et al., 2010). Produced by specific LAB strains, β-glucans contribute to a slimy or viscous consistency during fermentation and have demonstrated potential as stabilizers due to their rheological properties (Garai-Ibabe et al., 2010). Microbial β-glucans also offer beneficial health effects, including prebiotic (Palencia et al., 2009), anti-inflammatory (Notararigo et al., 2021), and immunomodulatory activities (Notararigo et al., 2014), as proven for the β-glucan producing strain *Pediococcus parvulus* 2.6. β-Glucan producing strains have previously been reported for different LAB species including *P. parvulus* (Dueñas-Chasco et al., 1997), *Paucilactobacillus suebicus* (Garai-Ibabe et al., 2010), *Oenococcus oeni* (Dols-Lafargue et al., 2008) and *Levilactobacillus brevis* (Fraunhofer et al., 2018). The suitability and safety of β-glucan-producing strains, such as *Lv. brevis* TMW 1.2112 and *P. parvulus* LTH 1110, for the use as food starter cultures have recently been demonstrated (Zipori et al., 2025).

Fermentative *in-situ* production of EPS by LAB has been applied successfully in various food matrices. For example, β-glucan-producing strains such as *Lv. brevis* TMW 1.2112 and recombinant *Lacticaseibacillus paracasei* NFBC 338 improved viscosity, texture, and sensory characteristics in sourdough and yogurt, respectively (Bockwoldt et al., 2021; Kearney et al., 2011). Fermentation of carrot puree with the β-glucan-forming strain *Pediococcus claussenii* ATCC BAA-344 led to enhanced elasticity and structural integrity due to β-glucan production (Juvonen et al., 2015). Extending this approach to fruit-based systems, particularly fruit purees, presents challenges due to their low pH-value, which inhibits the growth of many LAB strains (Garcia et al., 2016). Compared to fruit preparations, which are more highly processed products and contain added sugar and stabilizers, fruit purees are low processed and mostly consist of raw fruit or fruit parts, which were merely broken up and pasteurized. Therefore, fruit purees can be a hostile environment for bacteria, especially due to their high acidity and comparatively low sugar content. Some LAB strains can adapt and grow in such acidic environments, but achieving a level of EPS-mediated structural modification comparable to that observed in dairy systems has been limited so far (Guérin et al., 2020). Additionally, efforts to improve rheological properties through the incorporation of dried plant materials, such as fruit and vegetable pomaces, have only shown partial success in replacing conventional hydrocolloids. These materials often require blending with other stabilizers or processing modifications to achieve acceptable textural properties and stability (Karwacka et al., 2021, 2024; Viegas et al., 2024). A more promising strategy towards sustainable and consumer-friendly alternatives to structure-forming food additives lies in the use of fruit fermentates, which result from fermentation with LAB strains being able to produce EPS.

The aim of this study was to explore whether fermentates yielded by known EPS-producing LAB strains can have a stabilizing effect on fruit pieces when incorporated into model fruit preparations. For this purpose, it was investigated whether fermentation can convert peach puree into a structure-forming fermentate, which causes a rheologically detectable stabilizing effect when added to strawberry fruit preparations instead of conventional thickeners.

## 2 Materials and methods

### 2.1 Starting materials and chemicals

The peach puree used for incubation to produce the structure-forming fermentates and the strawberry puree used for the model fruit preparations had been produced industrially for this study. They were provided as numerous frozen aliquots of pasteurized purees, which were stored at −20 °C and thawed at 4 °C when required as starting material for experiments. Peach puree was selected as fermentation matrix, because in pretrials, it had been identified as the most promising substrate for fermentation. Among purees of peach, carrot, strawberry and beetroot, only the peach puree had led to a fermentatively induced increase in viscosity, which was rheologically measurable. The flow curves of those pretrial samples are displayed in Fig. S1 (cf. supplementary material).

Standard substances for the quantitation of free sugars and amino acids were acquired from Sigma Aldrich.

### 2.2 Bacterial strains and growth conditions

The LAB strains *Levilactobacillus brevis* TMW 1.2112 and *Pediococcus parvulu*s LTH 1110 were obtained from the strain collection of the Department of Food Microbiology and Hygiene of the University of Hohenheim. All strains were cultivated in de Man, Rogosa and Sharpe (MRS) medium (Merck KGaA, Darmstadt, Germany), without shaking, at 30 °C and under a modified gas atmosphere (10 % air, 10 % carbon dioxide, 80 % nitrogen) for 48–72 h.

### 2.3 Microbial analysis of fruit purees

Prior to their use for fermentation trials and processing (cf. 2.4), the fruit purees were subjected to microbial analysis to assess the possibility of contamination by autochthonous microbiota during the planned fermentation process. For this purpose, 10 g of peach puree was mixed with sterile 0.9 % sodium chloride solution (w/v) to a final weight of 100 g. The mixture was homogenized using a Stomacher homogenizer (Seward Ltd., Worthing, United Kingdom) for 60 s at 260 rpm. A serial decimal dilution of the homogenized sample up to a dilution of 10^−3^ was prepared, and 100 μL of each dilution step was plated in duplicates on Standard I agar (15 g/L peptone, 3 g/L yeast extract, 6 g/L NaCl, 12 g/L agar: pH: 7.5). Plates were incubated at 30 °C for 72 h to determine total aerobic mesophilic counts. The presence of yeasts and molds was assessed in the same manner, using yeast glucose chloramphenicol (YGC) agar (Merck KGaA) with incubation of the plates at 25 °C for five days. The detection limit in both analyses was 10 CFU/g. This testing was performed in two independent replicates.

### 2.4 Fermentation of peach puree and fermentate production

Fermentation of peach puree was carried out with either the heterofermentative strain *Lv. brevis* TMW 1.2112 or the homofermentative strain *P. parvulu*s LTH 1110. The production of β-glucan by *Lv. brevis* TMW 1.2112 has been previously confirmed (Fraunhofer et al., 2018). *P. parvulus* LTH 1110 is an EPS-producing strain, which is considered to be a β-glucan producer according to genetic and phenotypic evidences (Zipori et al., 2025).

Overnight cultures were prepared by inoculating a single colony of each strain into 10 mL of MRS broth and incubation under the conditions stated above (cf. 2.2). After incubation, the optical density at 600 nm (OD_600_) was measured to estimate the microbial counts (CFU/mL). Cultures were then centrifuged at 4000×*g* for 10 min at 4 °C. The supernatant was discarded, and the cell pellet was washed with 10 mL sterile 0.9 % (w/v) sodium chloride solution. After a second centrifugation step, the pellet was resuspended in 1 mL sterile 0.9 % (w/v) sodium chloride solution and adjusted to a final concentration of 10^9^ CFU/mL.

For subsequent fermentation, 150 mL of the starting peach puree (cf. 2.1) was transferred into a sterile 250 mL screw-lid glass jar. Inoculation was performed by adding 1.5 mL of the prepared bacterial suspension to achieve an initial microbial count of approximately 10^7^ CFU/mL. Uninoculated control samples were prepared by adding 1.5 mL of sterile 0.9 % NaCl solution without bacterial inoculum. Fermentation was conducted at 30 °C for 72 h under stationary aerobic condition in an incubator. Viable counts and pH values were determined at the beginning (t = 0 h) and at the end of the fermentation (t = 72 h). For pH measurement, 1 mL of thoroughly mixed fermentation sample was taken and analyzed using a pH meter (Testo 206, Testo SE & Co. KGaA, Germany). For microbial enumeration, 1 mL of the fermentation mixture was serially diluted in sterile 0.9 % sodium chloride solution and 100 μL of the last three dilution stages (10^−5^,10^−6^,10^−7^) were plated in duplicate on MRS agar. The plates were then incubated as described above. Colony-forming units (CFU/mL) were determined post-incubation.

**Fig. 1:**
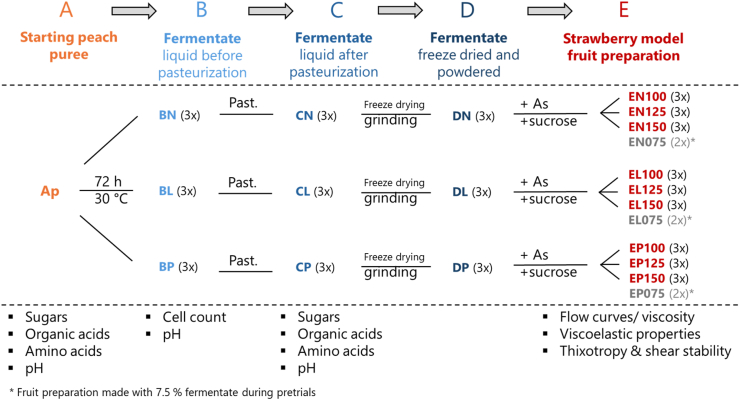
Experimental design: Production of peach fermentate (process steps B, C, D) from peach puree (Ap in step A) and its application as the sole stabilizer in the production of strawberry model fruit preparations (step E) from strawberry puree (As). Fermentate variants: N, uninoculated control (fermentate blank); L, fermented by *Levilactobacillus brevis* TMW 1.2112; P, fermented by *Pediococcus parvulus* LTH 1110. Analyses are listed below each process step.

To ensure that fermentation was terminated after 72 h, the fermentate was pasteurized at a core temperature of 80 °C for 3 min. The experiment was performed with three independent biological replicates (*n* = 3) for each variant, which comprised the peach fermentate blank (CN in Fig. 1), the *Lv. brevis* peach fermentate (CL), and the *P. parvulus* peach fermentate (CP).

The fermentates were lyophilized for 72 h in a Sublimator VaCo 10-D (Zirbus, Bad Grund, Germany) at 0.6 mbar and −25 °C for the first 62 h, 0.1 mbar and −30 °C for the next 6 h, and finally at 0.05 mbar and −20 °C for 4 h. The freeze-dried fermentate was ground in a centrifugal mill (ZM1, Retsch, Haan, Germany) equipped with a 0.5 mm sieve, yielding the powders of peach fermentate blank (DN), *Lv. brevis* peach fermentate (DL), and *P. parvulus* peach fermentate (DP) (Fig. 1). The powdered form was chosen for easy dosage and to mimic the application of conventional stabilizers (hydrocolloid powders) best possible. The whole process of peach fermentate production from the starting peach puree (process step A) across the process steps B, C, and D and final application as the sole stabilizer in the production of strawberry model fruit preparations (process step E) is illustrated in Fig. 1.

### 2.5 Production of fruit preparations

Strawberry model fruit preparations (process step E in Fig. 1) were produced on the laboratory scale. Per batch, 12 g of starting strawberry puree (cf. 2.1) was heated to a core temperature of 60 °C, afterwards the dry premix of lyophilized peach fermentate variant and granulated sucrose was added gradually, and the sample was stirred until the ingredients were completely dissolved. For subsequent pasteurization, the jar was placed into another water bath at 90 °C and held at this temperature for 10 min. Each fruit preparation consisted of 40 % strawberry puree, while the doses of each lyophilized peach fermentate variant varied (7.5, 10.0, 12.5, and 15.0 %). By adding the precalculated amount of granulated sucrose, the content of total soluble solids was always adjusted to a refractometer reading of 50 °Brix (i.e., 50 g/100 g), considering the amount of fermentate added and the total soluble solids content of the strawberry puree. This base recipe was adapted from the description by O'Rell and Chandan (O'Rell and Chandan, 2006) for standard fruit preparations for dairy products. Each variant of strawberry model fruit preparation was produced in triplicate (Fig. 1). Additionally, analogously produced products with fermentate doses of 7.5 %, which had been produced in duplicate during pretrials, were included. An image of a strawberry fruit preparation prepared in the study can be seen in the supplementary materials Fig. S2.

### 2.6 Chemical analysis of starting materials, incubated puree, and fruit preparations

#### 2.6.1 Quantification of sugar composition of starting fruit purees and fermentates

To prepare the samples for the analysis of free sugars, 20 mg of polyvinylpolypyrrolidone (PVPP) and 1 g of starting peach puree (Ap; Fig. 1) or pasteurized fermentate (CN, CL, CP) were diluted in 100 mL ultrapure water and centrifuged at 12,000×*g* and 4 °C (Avanti J26 XPI, Beckman Coulter, Brea, USA) for 10 min. From the clear supernatant, 10 mL was adjusted to the pH-value of 7, using 0.25 N NaOH, and further diluted 1:100 (final dilution of the samples 1:10,000). This diluted solution was filtered through a 0.45 μm polyamide membrane filter into a brown 2 mL vial.

**Fig. 2:**
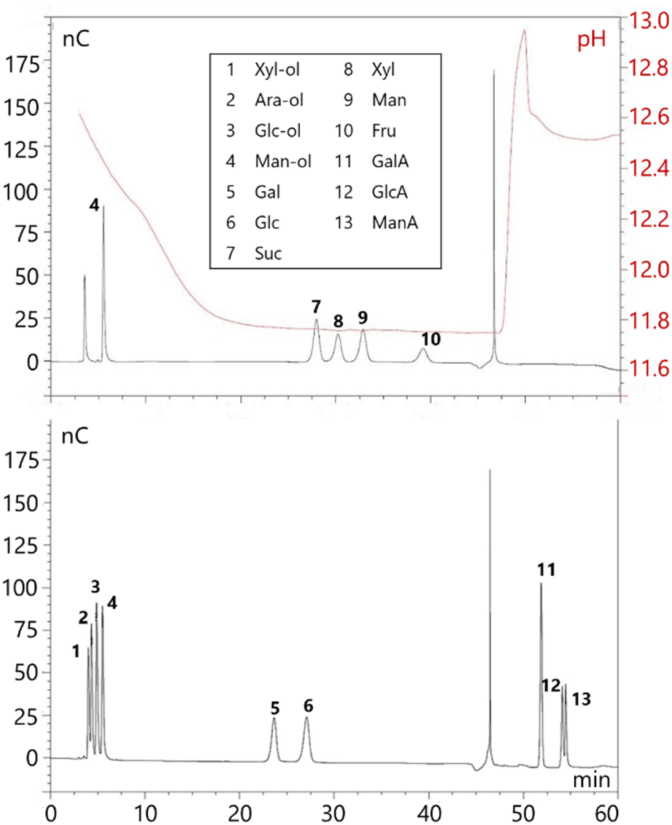
Chromatograms of mixed carbohydrate standards with pH gradient obtained with the HPAEC-PAD method involving gradient elution according to Table 1. Alditols: Xyl-ol, xylitol; Ara-ol, D-arabitol; Glc-ol, D-sorbitol; Man-ol, D-mannitol. Monosaccharides: Gal, D-galactose; Glc, D-glucose; Xyl, D-xylose; Man, D-mannose; Fru, D-fructose. Disaccharides: Suc, sucrose. Uronic acids: GalA, D-galacturonic acid; GlcA, D-glucuronic acid; ManA, D-mannuronic acid.

For the quantification of free sugars, a Dionex ICS 3000 HPAEC-PAD system with an AS50 Dionex autosampler (Thermo Fisher Scientific, Sindelfingen, Germany) was used. The method developed by Nagel et al. (2014) for the analysis of pectin hydrolysates was adapted for concurrent quantitation of the major alditols and free mono- and disaccharides in fruit products. As membrane-filtered (0.2 μm) eluents ultrapure water (A), 0.17 M sodium acetate in 0.1 M NaOH (B), and 0.2 N NaOH (C) were used for gradient elution (Table 1). The flow rate was 0.2 mL/min. Separation of mono- and disaccharides was achieved on a 3 × 150 mm CarboPac PA20 column (Thermo Fisher Scientific) connected with an upstream 3 × 30 mm AminoTrap plus a 3 × 30 mm CarboPac PA20 guard column (Thermo Fischer Scientific). The carbohydrates were detected using an ED pulsed-amperometric detector with a gold working electrode and an Ag/AgCl reference electrode. As displayed by the chromatograms of standard substances (Fig. 2), reliable separation of the four alditols and six mono- and disaccharides was feasible in the same run, besides three uronic acids. Thus, this method was applicable to monitor the metabolization of free sugars during fruit fermentation. The carbohydrate composition of starting purees and pasteurized fermentates (Ap, CN, CL, CP; Fig. 1) was quantitated in triplicate.

**Table 1:** Gradient and eluent composition used with the HPAEC-PAD System.

RT [min]	Eluent An Ultrapure water	Eluent B 0.17 M NaOAc in 0.1 M NaOH	Eluent C 0.2 M NaOH
−14	0	0	100
−12	98	2	0
0	98	2	0
Injection and start of linear NaOAc gradient			
3	99	1	0
39	0	100	0
51	0	0	100
60	0	0	100

#### 2.6.2 Determination of organic acid composition of fruit purees and fermentates

For both, the pasteurized fermentates (CN, CL, CP) and the starting peach puree (Ap; Fig. 1), the organic acid composition was determined enzymatically, using enzyme assay kits for D-/L-lactate, acetate, citrate, L-ascorbate, formate, L-malate, and ethanol (R-Biopharm, Pfungstadt, Germany) according to the manufacturer's protocol. For sample preparation, 1 g of puree or fermentate was diluted with ultrapure water to a final volume of 20 mL and then filtered through a pleated filter. All assays were performed in duplicate for each sample.

#### 2.6.3 Determination of amino acid composition of fruit purees and fermentates

For quantitation of free amino acids, an Acquity UPLC HClass system with vacuum degasser, Quaternary Solvent Manager, Sample Manager FTN, column heater, photodiode array detector, and Empower 3.8.1 software (Waters, Milford, USA) and the AccQ•Tag Ultra Derivatization Kit (Waters) were used as described by the manufacturer (Acquity UPLC H-Class and H-Class Bio Amino Acid Analysis System Guide, Revision B, 2012; Waters Corporation, 2024). Separation was achieved on a 2.1 × 100 mm AccQ•TAG ULTRA C18 1.7 μm column (Waters) at 43 °C and detected at 260 nm (Waters Corporation, 2024) following the standard gradient conditions described by Waters (Waters Corporation, 2024). The injection volume (20 °C) was 10 μL. For calibration based on norvaline as internal standard, both the mixed amino acid hydrolysate standard (Waters) and the respective membrane-filtered (0.45 μm) single standard solutions of the amino acids (250 μM each) aspartic acid (Asp), alanine (Ala), arginine (Arg), cystine (Cys, 125 μM), glutamic acid (Glu), histidine (His), isoleucine (Ile), leucine (Leu), lysine (Lys), methionine (Met), phenylalanine (Phe), proline (Pro), serine (Ser), tyrosine (Tyr), valine (Val), glycine (Gly), and threonine (Thr) were used (Waters Corporation, 2024). By analogy, internal standard calibration was extended by including asparagine (Asn), glutamine (Gln), ornithine (Orn), tryptophane (Trp), and γ-aminobutyric acid (GABA) as well as the biogenic amines putrescine, cadaverine, agmatine, and histamine. The limits of detection (LOD) and quantification (LOQ) were 0.5 μmol/L and 1 μm/L, respectively (Martin et al., 2023).

Approximately 20 mg of PVPP and 1 g of starting peach puree (Ap in Fig. 1) or pasteurized fermentate (CN, CL, CP in Fig. 1) were diluted in ultrapure water. The solution was made up to 20 mL and centrifuged at 12,000×*g* and 4 °C (Avanti J26 XPI, Beckman Coulter) for 10 min. From the clear supernatant, 2 mL was transferred into a 5 mL volumetric flask with 0.5 mL of 2.5 mM norvaline (internal standard). This solution was made to 5 mL with ultrapure water and filtered through a 0.45 μm-membrane filter into a vial. Precolumn derivatization of the analytes in both sample and standard solutions with aminoquinolyl-*N*-hydroxysuccinimidyl carbamate (AQC) was according to the instructions of the AccQ•Tag Ultra Derivatization Kit (Waters Corporation, 2024) with minor adaptation of handling. Borate buffer (70 μL), 10 μL of sample or standard solution and 20 μL of 10 mM AQC in acetonitrile were transferred into a 2 mL Eppendorf tube, which was vortexed for 10 s and kept at room temperature for 1 min prior to heating at 55 °C for 10 min. The heated solution was transferred into a brown 2 mL vial containing a 300 μL micro insert (WICOM Germany, Heppenheim, Germany). The capped vial was placed into the autosampler (20 °C), and injection followed immediately. Each standard and sample solution were analyzed in triplicate. For minor amino acids with partially non-detectable values, means were calculated using the TRIMMEAN function (Microsoft Excel 365 MSO, version 2506).

### 2.7 Rheological measurements

All rheological measurements were performed using a stress-controlled CVO 120 rotational rheometer (Bohlin Instruments, Pforzheim, Germany). Strawberry model fruit preparations from process stage E (Fig. 1), were measured at 20 °C. Samples were loaded with a spatula to ensure complete coverage of the gap between the stationary and mobile parts of the geometry. To prevent dehydration during analysis, the geometry was covered with a non-contacting plate equipped with a moist sponge.

#### 2.7.1 Flow curves and viscosity of model fruit preparations

Flow curves of the strawberry model fruit preparations were recorded at 20 °C in the rotational mode, using a plate/cone 4°/40 mm geometry (Bohlin Instruments) set to the standard gap size of 150 μm. Viscosity (*η*) was measured at 11 different shear rates ($\dot{\gamma }$), while $\dot{\gamma }$ was logarithmically increased from 0.08 s^−1^ to 590 s^−1^. The delay time between two measurement points was 100 s, while the integration time for each point was 40 s. The initial delay time was 120 s for thermal equilibration and relaxation after loading the sample. The total time per run was ∼28 min. Every fruit preparation variant (product) was produced three times and analyzed in triplicate.

#### 2.7.2 Viscoelastic properties of model fruit preparations

To analyze the viscoelastic properties of the strawberry model fruit preparations, a frequency sweep was recorded at 20 °C non-destructively in the oscillation mode, using a 40 mm plate/plate geometry (Bohlin Instruments) with a gap of 1000 μm. The storage (*G′*) and loss (*G″*) moduli were measured at 10 different oscillation frequencies and a target strain amplitude (*γ*) of 0.008, while the frequency (*f*) was logarithmically increased from 0.01 Hz to 10 Hz. The initial delay time was 120 s for thermal equilibration and relaxation after loading the sample. Every fruit preparation (product) was analyzed in triplicate.

#### 2.7.3 Rheological assessment of thixotropy and shear stability of model fruit preparations

To evaluate thixotropy of the strawberry model fruit preparations, the loaded sample underwent the following series S_1_ of consecutive rheological tests at 20 °C in a plate/plate 40 mm geometry with a gap of 1000 μm (Bohlin instruments): The initial non-destructive frequency sweep (FS) described in 2.7.2 (FS_0_) was immediately followed by (*a.*) a destructive shear step in the rotational mode (SS_1_) to break up the structure, (*b.*) another non-destructive frequency sweep (FS_S1_) to assess the remaining structure, (*c.*) a non-destructive time sweep (TS_1_) in the oscillation mode for recovery of the structure, and (*d.*) a final non-destructive frequency sweep (FS_R1_) to assess the recovered structure and thus thixotropy. To assess shear stability, analysis was continued by performing an identical second test series S_2_ with the same sample loading without interruption (sequences (*e.*) SS_2_, (*f.*) FS_S1_, (*g.*) TS_2_, (*h.*) FS_R1_). This sequence was terminated by (*k.*) a final amplitude sweep (AS) to verify previous non-destructive analysis and the linear-viscoelastic range of the strain amplitude (*γ*). The total run time of the entire job stream, covering both cycles S_1_ and S_2_ from FS_0_ to AS, was ∼2 h. By means of the frequency sweeps after each shear step (SS_*i*_) and each recovery step (TS_*i*_), the changes in the viscoelastic properties were determined after prior destruction (FS_S1_, FS_S2_) and recovery (FS_R1_, FS_R2_) relative to the starting state (FS_0_). For this purpose, the storage moduli *G*′(*f*_*j*_) of FS_S*i*_ and FS_R*i*_ were related to *G*′(*f*_*j*_) of FS_0_ for each of the frequencies *j* = 0.01 Hz, 0.1 Hz, 1 Hz and 10 Hz and expressed as percentage. The frequency sweeps FS_S1_, FS_R1_, FS_S2_, and FS_R2_ were performed as described in 2.7.2 for FS_0_, but always without the initial equilibration delay of 120 s. For each shear step (SS_1_, SS_2_), a constant shear stress (*τ*) of 100 Pa was continuously applied to measure the strain (and viscosity) seven times in the rotational mode with delay times of 10 s, integration times of 10 s, and waiting times of 10 s. The oscillatory time sweeps (TS_1_, TS_2_) were performed at a frequency *f* of 1 Hz and a target strain amplitude of *γ* = 0.008 (deduced from a starting stress of 1 Pa) to measure G′ and G″ 45 times at a delay time of 10 s and a waiting time of 10 s. The final amplitude sweep AS was performed at 1 Hz, recording G′, G″, and the strain (*γ*) 30 times with an initial delay time of 10 s, while the shear stress *σ* was logarithmically increased from 0.6 Pa to 60 Pa. Every fruit preparation (product) was analyzed in triplicate.

### 2.8 Statistical analysis

Using OriginPro 2025 software (OriginLab Corporation, Northampton, MA, USA), analysis of variance (ANOVA) with subsequent Tukey's post hoc test was applied to assess significant differences among group means, with a significance level of α = 0.05. For minor amino acids, Scheffé’s test was indicated instead of the Tukey's test (α = 0.05).

## 3 Results and discussion

### 3.1 Bacterial growth during fermentation and its effects on fruit puree composition

Prior to fermentation, the presence of microorganisms in the starting puree was assessed (Section 2.3) to exclude potential contamination during incubation. No colonies of aerobic mesophilic bacteria nor of yeasts and molds were detected in all replicates after 5 days of incubation. In both cases, the detection limit was 10 CFU/g. This finding indicated a minimal risk of spontaneous microbial growth that could interfere with the fermentation process, particularly from molds and yeasts, which typically thrive in sugar-rich substrates and can tolerate acidic environments (Tournas et al., 2006).

To evaluate the performance of EPS-producing LAB in a fruit-based matrix, peach puree was fermented under controlled conditions by either of the strains *Lv. brevis* TMW 1.2112 and *P. parvulus* LTH 1110 and compared with an uninoculated control sample (fermentate blank). Microbial viability, growth dynamics, and metabolic activities affecting the chemical composition of the matrix were determined. The viable counts of the LAB strains as well as the pH values of the peach puree before and after the incubation time are listed in Table 2.

**Table 2:** Viable counts and pH values of the fermentates at the beginning (0 h) and the end (72 h) of the fermentation.

Strain	Time [h]	Viable Count [10^7^ CFU/mL]	pH [-]
*Lv. brevis* TMW 1.2112 (BL)^*i*^	0 h	3.9 ± 0.9^A^	3.66 ± 0.02^A^
	72 h	12 ± 2.0^B^	3.78 ± 0.02^B^
*P. parvulus* LTH 1110 (BP)	0 h	1.5 ± 0.0^C^	3.66 ± 0.02^A^
	72 h	3.9 ± 0.4^AC^	3.77 ± 0.01^B^
Uninoculated control (BN)	0 h	–	3.66 ± 0.01^A^
	72 h	–	3.65 ± 0.02^A^

Mean ± standard deviation of 3 biological replicates. Different superscript capital letters in the same column refer to significant differences (*p* ≤ 0.05) between means.

*^i^*Liquid peach fermentate samples BL, BP and BN directly after fermentation, but prior to pasteurization (cf. Fig. 1).

As shown in Table 2*, Lv. brevis* TMW 1.2112 grew in the matrix by approximately one logarithmic unit from 3.9 ·10^7^ to 1.2 ·10^8^ CFU/mL during the 72 h of fermentation. In contrast, the viable counts of *P. parvulus* LTH 1110 remained stable throughout, indicating survival in this fruit matrix without significant growth. A slight but statistically significant increase in pH-value (from 3.66 to 3.78 for *Lv. brevis* TMW 1.2112) was observed after 72 h in all fermented samples (Table 2), indicating metabolic activity. Uninoculated controls did not show such a change, confirming that microbial metabolism was responsible for the pH changes indicated above.

By chemical analysis of the starting peach puree (Ap, cf. Fig. 1) and the resulting pasteurized fermentates (CN, CL, CP in Fig. 1), the effects of the two LAB strains on the food matrix were evaluated. The molar changes in the patterns of low-molecular carbohydrates and organic acids (Table 3) were indicative of the C-sources consumed and the produced metabolites, and thus of the fermentation pathways. As free amino acids represent a major N-source in fruit, the amino acid patterns before and after fermentation were compiled in Table 4.

**Table 3:** Composition of starting peach puree and resulting peach fermentates: Total soluble solids (refractometrically) and molar contents of free sugars, organic acids, and ethanol.

	Starting puree	Pasteurized fermentate		
	Ap Not incubated	CN Uninoculated	CL *Lv. brevis* TMW 1.2112	CP *P. parvulus* LTH 1110
Total soluble solids [g/100 g]	11.7 ± 0.01	11.6 ± 0.3	11.4 ± 0.1	11.6 ± 0.2

*Molar contents (f.w.)*				
Sorbitol [mmol/kg]	15.8 ± 0.2 ^A^	17.8 ± 2 ^A^	17.2 ± 1 ^A^	16.1 ± 1 ^A^
Mannitol [mmol/kg]	n.d.	n.d.	15.7 ± 1.7	n.d.
Glucose [mmol/kg]	158.8 ± 1 ^A^	120.7 ± 4 ^B^	111.7 ± 7 ^B^	113.2 ± 9 ^B^
Sucrose [mmol/kg]	97.7 ± 1 ^A^	76.8 ± 7 ^B^	75.1 ± 4 ^B^	75.2 ± 13 ^B^
Fructose [mmol/kg]	146.6 ± 2 ^A^	127.8 ± 10 ^AB^	109.8 ± 8 ^B^	121.6 ± 16 ^AB^
Total carbohydrates^i^ [mmol/kg]	418.8 ± 5 ^A^	343.0 ± 17 ^B^	328.4 ± 17 ^B^	326.0 ± 38 ^B^
Acetate [mmol/kg]	0.2 ± 0.3 ^A^	1.1 ± 0.9 ^A^	14.0 ± 2.2 ^B^	1.35 ± 0.1 ^A^
D-Lactate [mmol/kg]	12.2 ± 5.0 ^AB^	7.9 ± 2.7 ^AB^	13.6 ± 3.2 ^B^	5.7 ± 0.3 ^A^
L-Lactate [mmol/kg]	n.d.	1.8 ± 3.0 ^A^	32.9 ± 4.1 ^C^	20.2 ± 4.4 ^B^
L-Ascorbate [mmol/kg]	n.d.	n.d.	n.d.	n.d.
L-Malate [mmol/kg]	18.0 ± 0.9 ^A^	22.8 ± 8.0 ^A^	1.1 ± 1.9 ^B^	9.6 ± 4.2 ^AB^
Citrate [mmol/kg]	18.5 ± 1.4 ^A^	6.8 ± 1.8 ^B^	5.3 ± 2 ^B^	n.d.
Ethanol [mmol/kg]	0.2 ± 0.2 ^AB^	0.3 ± 0.2 ^AB^	0.9 ± 0.2 ^C^	0.8 ± 0.2 ^BC^

Mean ± standard deviation of three biological fermentate replicates, which were analyzed in triplicate each. Different superscript capital letters in the same row refer to significant differences (*p* ≤ 0.05) between means. n.d., not detectable; f.w., based on fresh weight. ^i^ Calculated as the sum of the listed carbohydrates that were detected individually.

**Table 4:** Amino acid composition of starting peach puree and resulting pasteurized peach fermentates.

Molar content (f.w.) of free amino acid	Starting puree	Pasteurized fermentate		
	Ap Not incubated	CN Uninoculated	CL *Lv. brevis* TMW 1.2112	CP *P. parvulus* LTH 1110
Asn [μmol/kg]	318.0 ± 4.4 ^A^	305.6 ± 9.9 ^A^	289.8 ± 36.5 ^A^	287.0 ± 32.4 ^A^
Gly [μmol/kg]	20.8 ± 0.01 ^A^	n.d.	n.d.	n.d.
Ser [μmol/kg]	12.5 ± 0.1 ^A^	6.9 ± 0.6 ^B^	6.1 ± 1.9 ^B^	6.6 ± 1.6 ^B^
Glu [μmol/kg]	12.2 ± 1.5 ^A^	5.3 ± 1.1 ^A^	n.d.	4.3 ± 0.8 ^A^
Asp [μmol/kg]	5.9 ± 5.2 ^A^	6.1 ± 1.1 ^A^	5.9 ± 1.7 ^A^	6.7 ± 0.9 ^A^
Ala [μmol/kg]	5.1 ± 0.02 ^A^	5.2 ± 0.4 ^A^	3.8 ± 0.8 ^A^	4.7 ± 0.8 ^A^
Thr [μmol/kg]	3.6 ± 0.01 ^A^	3.3 ± 0.4 ^AB^	2.4 ± 0.7 ^B^	2.8 ± 0.4 ^AB^
Val [μmol/kg]	2.6 ± 0.02 ^A^	2.0 ± 0.3 ^AB^	1.6 ± 0.5 ^B^	1.8 ± 0.2 ^AB^
Pro [μmol/kg]	n.d.	1.4 ± 0.2 ^A^	1.7 ± 0.5 ^A^	1.7 ± 0.2 ^A^

Mean ± standard deviation of three biological fermentate replicates, which were analyzed in triplicate each. Different superscript capital letters in the same row refer to significant differences (*p* ≤ 0.05) between means. n.d., not detectable; f.w., based on fresh weight.

For the starting puree (Ap), the contents of the major sugars (glucose, fructose, sucrose, sorbitol) and acids (citrate, L-malate) were found to be within the typical ranges for peach according to the AIJN Code of Practice (European Fruit Juice Association (AIJN)). Compared to Ap, the total carbohydrate content of the pasteurized fermentate blank (uninoculated control, CN) had declined by 18 %, which was mainly due to significant decreases of glucose (by 24 %), besides sucrose (by 21 %) and fructose (by 13 %). Accordingly, mere incubation (30 °C, 72 h) and the subsequent pasteurization of the CN sample caused a carbohydrate loss that was irrespective of induced LAB growth. As minor dilution of the puree due to the addition of the blank inoculum was negligible (cf. 2.4), losses of sugars may mainly be ascribed to partial sucrose hydrolysis and the involvement of reducing sugars in Maillard reaction during post-fermentative pasteurization (Louarme and Billaud, 2012; Yıkmış et al., 2023). However, although the risk of interfering spontaneous microbial growth during incubation had been rated negligible (cf. 2.3), minor microbial metabolism of citrate during incubation of the control batches (CN) could not be excluded entirely, as suggested by the citrate loss by 63 % relative to Ap and the associated minor increases in acetate and L-lactate. In this context, the slightly elevated D-lactate content of Ap should be noted as well (European Fruit Juice Association (AIJN)). Nevertheless, even post incubation, no microorganisms were isolated from the uninoculated purees, using the method in section 2.3.

Although inoculation had yielded strikingly high initial microbial counts (>10^7^, Table 2), fermentation by *Lv. brevis* TMW 1.2112 and by *P. parvulus* LTH 1110 resulted in pasteurized peach fermentates CL and CP which did not differ significantly from the fermentate blank CN in terms of both total soluble solids and total carbohydrate contents (Table 3). Likewise, the three fermentate variants displayed insignificantly different patterns of mono- and disaccharides. Especially sucrose and sorbitol were unaffected. However, compared to the starting puree Ap, the significant losses of glucose and fructose were greatest for the *Lv. brevis* TMW 1.2112 fermentates CL (by 30 % and 25 %, respectively). Most importantly, mannitol was only found in CL. This was consistent with the presence of a mannitol dehydrogenase gene in its genome (Zipori et al., 2025), which catalyzes the reduction of fructose to mannitol, a pathway being typical of heterofermentative LAB (Weymarn et al., 2002).

Unlike the overall weak changes in carbohydrates, substantial shifts were observed in organic acid and ethanol profiles, especially for the CL fermentates. Fermentation by *Lv. brevis* TMW 1.2112 resulted in the strongest lactate production (4.1 g/kg), yielding L- and D-lactate contents of 33 and 14 mmol/kg CL, which corresponded to 18- and 1.7-fold increases compared to the fermentate blank CN (Table 3). Likewise, acetate formation was strongest for CL (13-fold increase up to 14 mmol/kg, i.e., 0.84 g/kg). The 3-fold increase in ethanol was significant, but the ethanol content of 0.04 g/kg was irrelevant, although maximum among the three fermentate variants. These metabolites are typical products of heterofermentative metabolism in LAB and indicate active conversion of hexoses or pentoses into acids and alcohols via the phosphoketolase pathway (Gänzle, 2015). Lactate production by *P. parvulus* LTH 1110 was also pronounced (3.3 g/kg), yielding L- and D-lactate contents of 20 and 6 mmol/kg CP. Thus, there was an 11-fold increase in L-lactate for CP, while the contents of D-lactate, acetate, and ethanol were insignificantly different from the fermentate blank CN, overall consistent with the homofermentative metabolism.

Characteristic of both strains was the strong depletion of L-malic acid (21- and 2.4-fold decrease for CL and CP compared to CN, Table 3), supporting the occurrence of malolactic fermentation. The L-malate content decreased from 3.1 g/kg CN to 1.3 g/kg CP and only 0.15 g/kg CL. This reduction, along with elevated L-lactate levels, indicated a metabolic shift in response to low pH-value stress, a phenomenon commonly observed in LAB during wine fermentation (Wang et al., 2018). Genomic analysis supported this, with both strains carrying genes encoding the malolactic enzymes (*Lv. brevis* TMW 1.2112: AZI09_00840; *P. parvulus* LTH 1110: ABUE38_01020). Peach puree typically contains almost equal amounts of L-malate and citrate (Table 3, (European Fruit Juice Association (AIJN))). Another common feature of both LAB strains was the strong decline of citrate. The citrate content fell to 1 g/kg in CN and even to an undetectable level in CP. While L-malate was most degraded by *Lv. brevis* TMW 1.2112, citrate degradation was strongest for *P. parvulus* LTH 1110. This observation might highlight the role of citrate metabolism as a key adaptive strategy employed by LAB to survive and maintain cellular homeostasis under acidic conditions. In such environments, the uptake and catabolism of citrate support bacterial energy metabolism and contribute to intracellular pH regulation through proton-consuming decarboxylation reactions (Eicher et al., 2023; Hugenholtz, 1993). The strong reduction in the contents of L-malate and citrate were consistent with the slight increase in pH-values in the fermentates.

Both in the peach puree Ap and in the fermentates CN-CP, eight different free amino acids, but no biogenic amines were found (Table 4). Asparagine always predominated, constituting >80 % of the total free amino acids, whereas the other amino acids were mostly rather close to LOQ. This pattern is compliant with the reference guideline of the AIJN Code of Practice for peach puree and juice (European Fruit Juice Association (AIJN)), although the contents found were substantially lower. However, it is known that the contents of free amino acids can vary greatly depending on cultivar, crop year, and processing technology. It should be noted that no sulfur-containing and no aromatic amino acids were detected. For peach puree, methionine and phenylalanine were reported to be typically among the minor amino acids (European Fruit Juice Association (AIJN)).

As observed for the free sugars, the amino acid composition of the uninoculated control fermentate (CN) slightly differed from that of the puree prior to processing (Ap). Due to this blank fermentation step with subsequent pasteurization, the low glycine, serine, and glutamic acid contents of the puree declined to either undetectable levels or contents rather close to LOQ (Table 4). These changes might be ascribed to the involvement of amino acids in Maillard reaction during pasteurization. However, the predominant amino acid Asn was insignificantly affected by this treatment. Proline usually occurs at very low contents in peach puree (European Fruit Juice Association (AIJN)), but was not detected in AP. Consistently, proline levels found in the control fermentates CN were very close to the LOQ.

Compared to the blank fermentate CN, fermentation by *Lv. brevis* TMW 1.2112 and by *P. parvulus* LTH 1110 hardly affected the free-amino acid pattern. Apart from undetectable glutamic acid in the CL fermentates, the amino acid patterns of the blank fermentates CN and those of the CL and CP fermentates were insignificantly different. This applied both to the predominant amino acid asparagine and to the minor ones (serine, aspartic acid, alanine, threonine, valine, and proline), indicating their limited involvement in microbial metabolism under the test conditions. No biogenic amines were detected in any of the fermented samples, which is a positive safety indicator. Accordingly, the LAB strains used did not activate decarboxylation pathways that convert amino acids into potentially harmful amines, which is otherwise a common stress response to acidic environment in some LAB (Barbieri et al., 2019).

Overall, the expected fermentation-induced changes of metabolites were detectable by compositional analysis of the fermentate variants. However, the occurring changes of the fruit matrix in terms of carbohydrates, amino acids, and pH value were low or even negligible. The pronounced losses of malate and citrate were most striking and aligned with known metabolic responses of LAB to acid stress. Under low pH conditions, LAB often shift from hexose fermentation to alternative pathways such as amino acid catabolism and malolactic fermentation, mechanisms that help to maintain redox balance and intracellular pH homeostasis (Gänzle, 2015). These stress-adaptive mechanisms likely contributed to the limited microbial growth and the moderate metabolic impact observed in the peach puree. The results in Table 2, Table 3, Table 4 suggest that thermal processing played a more prominent role in altering amino acid and sugar contents, likely due to Maillard reactions, rather than microbial activity. Additionally, since free sugars are the substrates used by the cells for EPS production, the low sugar consumption did not suggest major EPS formation by the strains.

The fact that the overall impact of fermentation on the biochemical composition was mild was consistent with the known potential of LAB to modulate food quality through subtle metabolic shifts. Many LAB strains are valued for their ability to produce desirable flavor compounds and textural changes in fermented products (Hu et al., 2022). As such, the moderate biochemical changes observed here may be beneficial from a sensory point of view. However, to fully assess the sensory impact and consumer acceptance of the fermented product, a comprehensive sensory evaluation is necessary. A follow-up study including a representative sensory panel is already planned to address this essential aspect for broader application and product development.

### 3.2 Structure-altering effect of fermentate addition to fruit preparations

#### 3.2.1 Effect of lyophilized fermentates on the viscoelastic behavior of fruit preparations

**Fig. 3:**
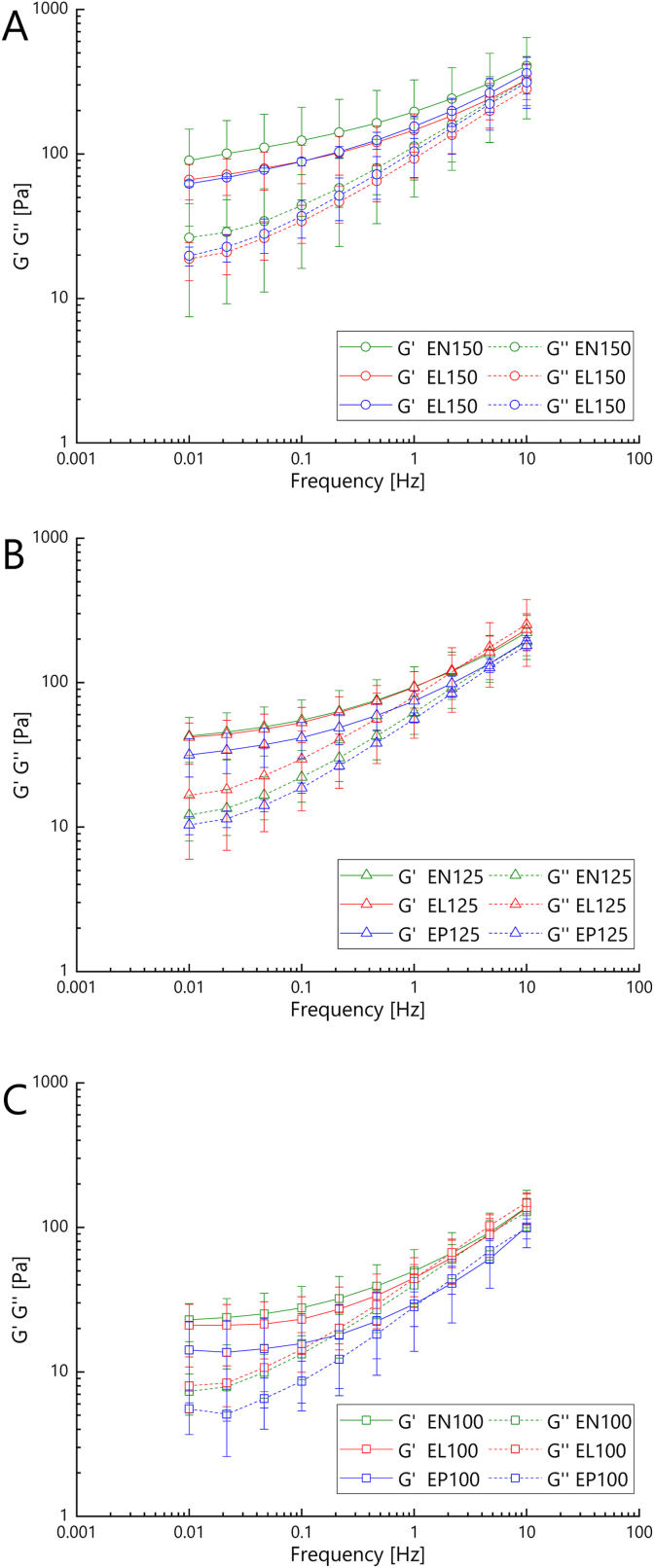
Viscoelastic properties of strawberry model fruit preparations containing different doses of lyophilized peach fermentate as the sole stabilizer: **A**, 15 %; **B**, 12.5 %; **C**, 10 % (e.g., EN150, EN125 and EN100 contained doses of 15 %, 12.5 % and 10 % of fermentate variant DN, respectively). Fermentate variant used in EN: DN, uninoculated control (fermentate blank, green); in EL: DL, *Lv. brevis* TMW 1.2112 fermentate (red); in EP: DP, *P. parvulus* LTH 1110 fermentate (blue). Storage modulus (*G*′(*f*)) and loss modulus (*G*''(*f*)) as a function of oscillation frequency (*f*) at 20 °C.

Viscoelastic behavior is of importance for fruit preparations for various technological reasons. They require high viscosity and gel-like properties when being in an idle state to stabilize fruit pieces by preventing their sedimentation or floating. Non-destructive rheological analysis of the strawberry model fruit preparations revealed their viscoelastic properties by monitoring the storage and the loss modulus as a function of oscillation frequency (*G*′(*f*) and *G*''(*f*), respectively; Fig. 3). The strawberry model fruit preparations always contained one of the three lyophilized fermentates (DN, DL, DP, cf. Fig. 1) as the sole stabilizer, while the stabilizer dose had been varied in a range of 10 %–15 % (nine fruit preparation variants, cf. Fig. 1).

Among the nine product variants and 27 model fruit preparation batches, all variants except one showed a higher value for the storage modulus (*G′*(*f*)) than for the loss modulus (*G''*(*f*)) throughout, irrespective of type and dose of fermentate (Fig. 3). Accordingly, their behavior was more elastic than viscous, which implied gel-like viscoelastic structures. Both moduli markedly increased with rising frequency, with the loss modulus displaying a more pronounced dependency on frequency. This type of frequency dependency was indicative of weak gel-like behavior (Kahyaoglu and Kaya, 2003), involving strong macromolecular entanglements. Similar behavior was observed for fruit jams in other studies (Xin et al., 2011). The only exception was the model fruit preparation stabilized with 10 % *P. parvulus* LTH 1110 fermentate (EP100), where *G′*(*f*) was lower than *G''*(*f*) at frequencies *f* > 1 Hz (Fig. 3C), indicating a softer structure with less entanglements.

Different fermentate doses resulted in different viscoelastic behavior. Both moduli increased with rising fermentate doses, irrespective of the fact whether the fruit preparations were made with *P. parvulus* LTH 1110 fermentate (DP), *Lv. brevis* TMW 1.2112 fermentate (DL) or fermentate blank (DN). At the highest fermentate doses (Fig. 3A), G′(*f*) and G''(*f*) were in the range of conventionally stabilized food products like fruit jams (Xin et al., 2011) and food gels stabilized with pectins (Haghighi et al., 2011). This shows that lyophilized LAB peach fermentate can be used at elevated doses as a substitute for conventional stabilizers to reach similar viscoelastic properties. However, at every fermentate dose, it was always the uninoculated control DN that yielded the fruit preparations displaying the highest G'(*f*) among the three fermentate variants DN-DP. Hence, the stabilizing potentials of the *P. parvulus* LTH 1110 fermentates (DP) and *Lv. brevis* TMW 1.2112 fermentates (DL) were rather similar, but though slightly inferior to that of the fermentate blank. While the addition of the peach fermentate blank enabled sufficient structure formation in terms of viscoelasticity, there was apparently no additional benefit of fermentation.

#### 3.2.2 Effect of lyophilized fermentates on flow behavior and viscosity of fruit preparations

**Fig. 4:**
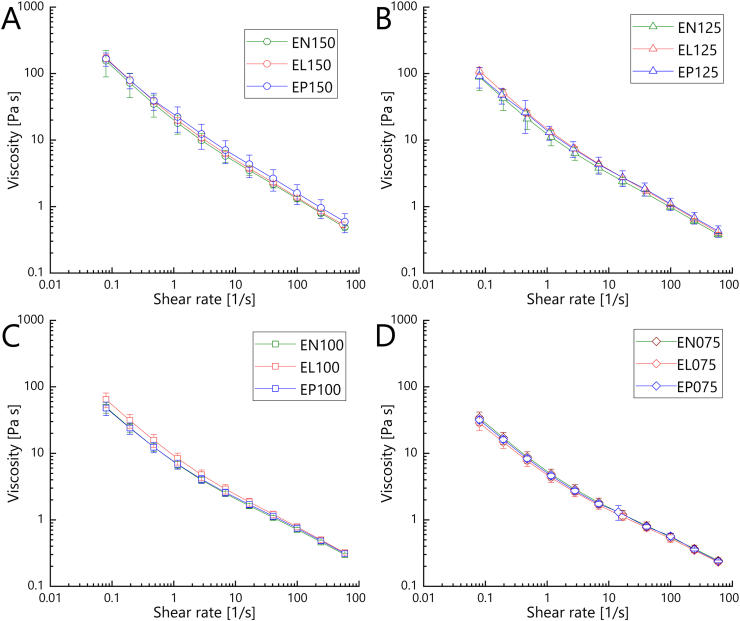
Flow curves (20 °C) of strawberry model fruit preparations containing different doses of lyophilized peach fermentate as the sole stabilizer: **A**, 15 %; **B**, 12.5 %; **C**, 10 %; **D**, 7.5 % (e.g., EN150, EN125, EN100 and EN075 contained doses of 15 %, 12.5 %, 10 %, and 7.5 % of fermentate variant DN, respectively). Fermentate variant used in EN: DN, uninoculated control (fermentate blank, green); in EL: DL, *Lv. brevis* TMW 1.2112 fermentate (red); in EP: DP, *P. parvulus* LTH 1110 fermentate (blue).

Flow behavior is of importance for fruit preparations for various technological reasons. Shear thinning behavior is desired to enable pumpability and stirring, but stabilization at idle state. Fig. 4 shows the flow curves, which were recorded for strawberry model fruit preparations that always contained one of the three lyophilized fermentates (DN, DL, DP, cf. Fig. 1) as the sole stabilizer, while the stabilizer dose was varied in a range of 7.5 %–15 % (twelve fruit preparation variants, cf. Fig. 1).

Irrespective of the fermentate type and dose, the model fruit preparations showed a decrease in viscosity with rising shear rate, and thus shear thinning behavior. This was compliant with reported findings of most studies dedicated to fruit preparations and met expectations (Meghwal et al., 2017). As revealed above by the frequency sweeps (3.2.1), a strong effect of the fermentate dose became also evident by the flow curves. The higher the dose of lyophilized fermentate was, the higher the viscosity of the model fruit preparation was, irrespective of whether the uninoculated control (DN) had been added or one of the two fermentate variants (DL, DP).

Aside from the predominant effect of the fermentate dose, a faint effect of the fermentate type became additionally evident for the fruit preparations containing 10 % *Lv. brevis* TMW 1.2112 fermentate (EL100 in Fig. 4C). Their viscosities were significantly higher than those of the fruit preparations containing 10 % of the uninoculated control fermentate. However, this fermentatively induced increase in viscosity was merely by factor 1.3, whereas enhancing the fermentate blank dose from 10 % to 15 % caused a 2.4-fold increase in viscosity of the strawberry model fruit preparations. Nonetheless, both effects were not comparable to the thickening potential of conventionally used stabilizers, which cause more than 10-fold increase in viscosity at doses <1 % (Sahin and Ozdemir, 2004; Meghwal et al., 2017).

#### 3.2.3 Effects of lyophilized fermentates on thixotropy and shear stability

Thixotropic behavior is an important property for fruit preparations, because they are affected by considerable shear forces during pumping and processing. A behavior being closer to a liquid while being in motion facilitates processability. In contrast, strong thickening resulting in a more gel-like behavior while being at idle state is important for sufficient stabilization of fruit pieces. It is essential that fruit preparations regain their original structure best possible after exposure to shearing and temporary structure loss. Moreover, recovery of the structure is necessary each time after repeated exposure to shearing. In addition to thixotropic behavior, high shear stability referring to recovery after repeated exposure to shear is thus a highly desirable property of fruit preparations (Meghwal et al., 2017). In the present study, thixotropic behavior was derived from a first shear-and-recovery cycle S_1_. Resistance to shear was indicated by the *G*'_S1_(*f*_*j*_)/*G*′_0_(*f*_*j*_) ratios after a first shear step, whereas recovery of the structure was specified by the *G*'_R1_(*f*_*j*_)/*G*′_0_(*f*_*j*_) ratios after a subsequent first recovery time (Table 5). Shear stability was deduced from a subsequent second shear-and-recovery cycle S_2_, yielding *G*'_S2_(*f*_*j*_)/*G*′_0_(*f*_*j*_) ratios after a second shear step and *G*'_R2_(*f*_*j*_)/*G*′_0_(*f*_*j*_) ratios after a second recovery time by analogy (Table 5). Comparisons were always relative to the original state (*G*′_0_(*f*_*j*_)), which is displayed in Fig. 3. Shear stability was thus defined as thixotropic behavior after multiple shear and recovery steps.

**Table 5:** Shear stability of strawberry model fruit preparation (MFP) variants containing different doses *c*_Ferm_ (10 %–15 %) of lyophilized peach fermentate as the sole stabilizer (e.g., EN150, EN125 and EN100 contained doses of 15 %, 12.5 % and 10 % of fermentate variant DN, respectively). Fermentate variant used in EN: DN, uninoculated control (fermentate blank); in EL: DL, *Lv. brevis* TMW 1.2112 fermentate; in EP: DP, *P. parvulus* LTH 1110 fermentate. Storage modulus (*G*′) and loss modulus (*G*″) as function of oscillation frequencies *f* at 20 °C after a first and a second shearing step (S1, S2) as well as after a first and a second recovery step (R1, R2), always relative to the original idle state.

*c*_Ferm_. [%]	MFP	*f*_*j*_ [Hz]	Relative resistance to shear		Relative recovery from shear	
			Cycle S_1_*G*'_S1_(*f*_*j*_)/*G*′_0_(*f*_*j*_) [%]	Cycle S_2_*G*'_S2_(*f*_*j*_)/*G*′_0_(*f*_*j*_) [%]	Cycle S_1_*G*'_R1_(*f*_*j*_)/*G*′_0_(*f*_*j*_) < [%]	Cycle S_2_*G*'_R2_(*f*_*j*_)/*G*′_0_(*f*_*j*_) [%]
**15.0**	EN150	0.01	115 ± 20	111 ± 19	140 ± 22	137 ± 20
		0.1	115 ± 20	112 ± 20	133 ± 25	131 ± 24
		1	112 ± 17	110 ± 17	124 ± 19	122 ± 19
		10	106 ± 10	103 ± 12	113 ± 12	111 ± 13
	EL150	0.01	114 ± 19	110 ± 18	134 ± 24	132 ± 24
		0.1	113 ± 20	110 ± 21	131 ± 25	128 ± 25
		1	112 ± 17	110 ± 17	124 ± 19	122 ± 19
		10	108 ± 11	105 ± 11	114 ± 12	112 ± 11
	EP150	0.01	106 ± 2^AB^	103 ± 3^A^	135 ± 11^B^	130 ± 9^B^
		0.1	107 ± 3	104 ± 1	126 ± 4	122 ± 1
		1	104 ± 4	101 ± 1	116 ± 4	113 ± 2
		10	100 ± 2	97 ± 1	106 ± 1	103 ± 1
**12.5**	EN125	0.01	125 ± 11	123 ± 10	141 ± 17	136 ± 10
		0.1	125 ± 13	121 ± 10	142 ± 13	140 ± 11
		1	118 ± 10	116 ± 8	131 ± 10	131 ± 9
		10	110 ± 4	109 ± 3	117 ± 5	116 ± 3
	EL125	0.01	118 ± 15	114 ± 15	122 ± 23	126 ± 21
		0.1	110 ± 16^B^	104 ± 20^A^	123 ± 19^A^	126 ± 18^A^
		1	112 ± 13	110 ± 12	125 ± 14	142 ± 32
		10	107 ± 8	105 ± 8	113 ± 9	112 ± 9
	EP125	0.01	122 ± 10	118 ± 11	137 ± 15	143 ± 18
		0.1	119 ± 13	115 ± 11	138 ± 18	135 ± 16
		1	114 ± 11	111 ± 10	127 ± 13	125 ± 11
		10	106 ± 4^AB^	103 ± 4^AB^	112 ± 4^B^	110 ± 4^AB^
**10.0**	EN100	0.01	128 ± 5	124 ± 12	141 ± 10	143 ± 6
		0.1	119 ± 7	117 ± 6	137 ± 7	136 ± 6
		1	116 ± 6	114 ± 5	128 ± 6	128 ± 4
		10	110 ± 3	107 ± 1	116 ± 3	114 ± 2
	EL100	0.01	116 ± 53	109 ± 44	134 ± 57	127 ± 51
		0.1	114 ± 50	107 ± 48	126 ± 58	120 ± 53
		1	114 ± 46	110 ± 44	126 ± 50	121 ± 49
		10	111 ± 26	105 ± 24	114 ± 26	110 ± 26
	EP100	0.01	126 ± 20	127 ± 29	146 ± 17	145 ± 22
		0.1	129 ± 29	127 ± 32	148 ± 37	148 ± 42
		1	129 ± 30	127 ± 34	142 ± 34	141 ± 39
		10	111 ± 11	109 ± 12	117 ± 14	115 ± 15

Mean ± standard deviation of three model fruit preparations made with three biological fermentate replicates, which were analyzed in triplicate each. Different superscript capital letters in the same row refer to significant differences (*p* ≤ 0.05) between means at different steps of the shear stability measurement. Means in rows without any superscript capital letters were not significantly different.

Irrespective of fermentate dose or type, the two shear steps hardly induced any measurable changes in structure, *G*'_S1_(*f*_*j*_)/*G*′_0_(*f*_*j*_) and *G*'_S2_(*f*_*j*_)/*G*′_0_(*f*_*j*_) remained closely around 100 %. Nonetheless, after each recovery phase, the storage moduli of every model fruit preparation were higher relative to the original state than right after the directly preceding shear step. Moreover, *G*'_R1_(*f*_*j*_)/*G*′_0_(*f*_*j*_) and *G*'_R2_(*f*_*j*_)/*G*′_0_(*f*_*j*_) were always rather similar and above 100 %, despite the lower *G*'_S2_(*f*_*j*_)/*G*′_0_(*f*_*j*_) after the second shear step between the two recovery periods. Consequently, thixotropic behavior was not recognizable, but high shear stability was proven throughout, irrespective of dose and type of fermentate. Consequently, the lyophilized peach fermentates were found to be applicable to produce highly shear-stable products. However, application of the two LAB strains did not result in a clear benefit compared to the fermentate blank. A structure-altering effect of due to fermentation by the LAB strains is not supported by the results of Table 5. However, it is important to note that there was also no negative rheological effect due to the use of these LAB strains for fermentate production.

## 4 Conclusion

This study demonstrated that lyophilized peach fermentates can serve as substitutes for structure-forming hydrocolloids, with the concept being technologically feasible in principle. Although survival and some metabolic activity of the two LAB strains in the acidic peach puree was possible, no measurable fermentation-induced rheological influence was observed. The ability of the strains to produce large amounts of EPS, which could be detected rheologically, appears to be limited due to apparent stress conditions at low pH values. A minor effect on flow behavior after fermentation with *Lv. brevis* TMW 1.2112 might indicate weak interactions between potentially formed EPS and fruit-native polysaccharides, but this effect was negligible compared to the influence of the fermentate blank dose. The effect of increasing doses of fermentate blank were much more pronounced and unambiguous. According to flow behavior and viscoelastic properties of the model fruit preparations, fermentate doses between 10 % and 15 % were needed, irrespective of the fermentate type. In terms of thickening properties at the idle state, the fermentate blank was found to be slightly superior to the two LAB fermentate variants at the highest dose. Achieved shear stability was striking throughout. Chemical analysis did not reveal any disadvantages or harm due to fermentation but supported the applicability of the fermentates as thickeners in fruit preparations. Overall, these findings suggested that fermentation with EPS-forming LAB does not significantly alter the rheological properties of fruit preparations under the given conditions, and that structure modification is primarily achieved through the addition of plant material rather than through metabolic changes by the LAB strains. Future studies should focus on understanding the metabolic mechanism of LAB strains in fermentation of fruit systems, in particular those involved in EPS formation, in order to allow maximization of their rheological effects on fruit systems.

## Author contributions

**Silvan Festini**: Food technology and chemical analysis: Conceptualization: (equal); Data curation (lead); Formal analysis (lead); Writing-original draft (lead). **Dor Zipori**: Fermentate production and microbiological analyses: Conceptualization (equal); Data curation (lead); Formal analysis (lead); Investigation (lead); Writing-original draft (lead). **Marc Wallisch**: Carbohydrate analysis by HPAEC-PAD: Investigation (lead); Methodology (supporting). **Agnes Weiss**: Conceptualization (equal); Writing-review & editing (equal); Funding acquisition (supporting). **Sybille Neidhart**: Conceptualization (equal); Methodology (supporting); Supervision (supporting); Writing-review & editing (equal); Funding acquisition (supporting). **Herbert Schmidt**: Funding acquisition (lead); Resources (equal); Supervision (equal); Writing-review & editing (equal). **Mario Jekle**: Funding acquisition (lead); Resources (equal); Supervision (equal); Writing-review & editing (equal).

## Ethical approval

Ethics approval was not required for this research.

## Data availability statement

Data is available on request from the authors. The bacteria strains used in this study are available upon request.

## Declaration of competing interest

The authors declare that they have no known competing financial interests or personal relationships that could have appeared to influence the work reported in this paper.
