# Supplementary material for: Structure formation in fruit preparations by fruit fermentates produced with exopolysaccharide-forming lactic acid bacteria
Source: Curr Res Food Sci. 2025 Nov 7;11:101244. doi: 10.1016/j.crfs.2025.101244 (PMC12657838; doi:10.1016/j.crfs.2025.101244)
Supplement: Multimedia component 1 [file mmc1.docx]

Structure formation in fruit preparations by fruit fermentates produced with exopolysaccharide-forming lactic acid bacteria

Silvan Festini, Dor Zipori, Marc Wallisch, Agnes Weiss, Sybille Neidhart, Herbert Schmidt, Mario Jekle

**Supplementary Data**

| **** | **** |
| --- | --- |
| **** | **** |

Figure S1 Flow curves (20 °C) of strawberry model fruit preparations containing different doses of lyophilized peach-, strawberry, carrot- or beetroot fermentate as the sole stabilizer. Fermentate variants used: Control = uninoculated control (fermentate blank, green); LB = Lv. brevis TMW 1.2112 fermentate (red); PP = P. parvulus LTH 1110 fermentate (blue)


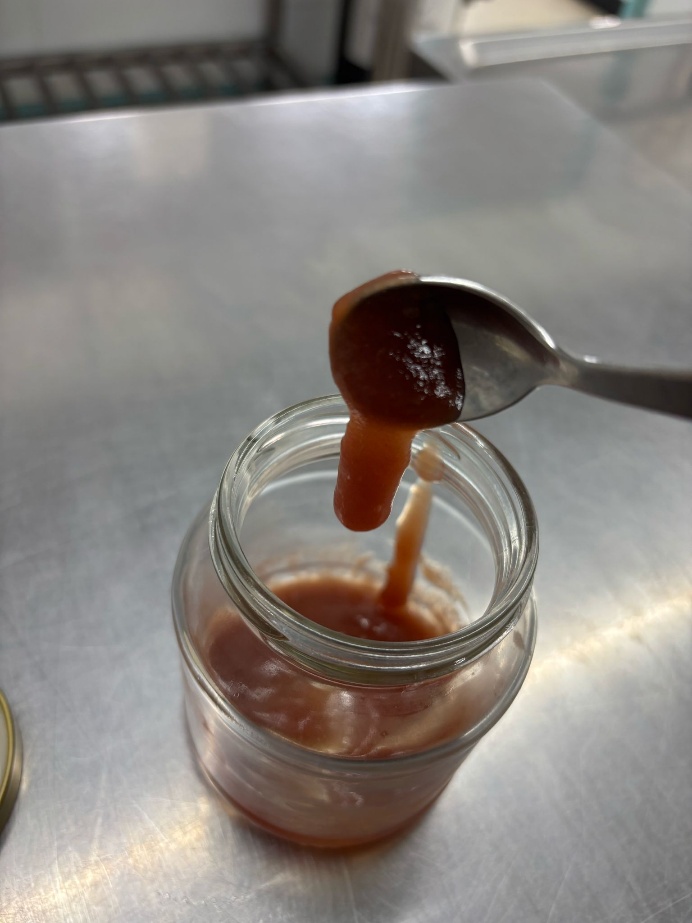


Figure S2 Photograph of a strawberry model fruit preparation made with 10.0% lyophilized Lv. brevis TMW 1.2112 peach fermentate as its sole stabilizer
